# Family-Based Cohort Association Study of *PRKCB1*, *CBLN1* and *KCNMB4* Gene Polymorphisms and Autism in Polish Population

**DOI:** 10.1007/s10803-021-05291-3

**Published:** 2021-09-25

**Authors:** Tomasz Iwanicki, Anna Balcerzyk, Beata Kazek, Ewa Emich-Widera, Wirginia Likus, Joanna Iwanicka, Agnieszka Kapinos-Gorczyca, Maciej Kapinos, Alicja Jarosz, Władysław Grzeszczak, Sylwia Górczyńska-Kosiorz, Paweł Niemiec

**Affiliations:** 1 Department of Biochemistry and Medical Genetics, School of Health Sciences in Katowice, Medical University of Silesia in Katowice, Medykow Street 18, 40-752 Katowice, Poland; 2 Child Development Support Center, Kępowa Street 56, 40- 583 Katowice, Poland; 3 Department of Pediatric Neurology, Faculty of Medical Science in Katowice, Medical University of Silesia in Katowice, Medykow Street 16, 40-752 Katowice, Poland; 4 Department of Anatomy, School of Health Sciences in Katowice, Medical University of Silesia in Katowice, Medykow Street 18, 40-752 Katowice, Poland; 5 CZP Feniks, Daily Ward for Children and Adolescents, Młyńska Street 8, 44-100 Gliwice, Poland; 6 Department of Internal Medicine, Diabetology, and Nephrology in Zabrze, Faculty of Medical Sciences in Zabrze, Medical University of Silesia in Katowice, 3-go Maja Street 13-15, 41-800 Zabrze, Poland

**Keywords:** Genetic testing, Protein kinase C beta 1 subunit gene, Single nucleotide polymorphism, Transmission-disequilibrium test

## Abstract

The aim of the study was to perform family-based association analysis of *PRKCB1*, *CBLN1* and *KCNMB4* gene polymorphisms and autism disorder. We comprised 206 Caucasian children with autistic spectrum disorder (ASD) and their biological parents. In transmission/disequilibrium test we observed that T-allele of the rs198198 polymorphism of the *PRKCB1* gene was more often transmitted to affected children in the male subgroup (p = 0.010). Additionally, the T carrier state was significantly associated with hypotonia (p = 0.048). In the female subgroup, the T-allele carriers more often showed more mobile/vital behavior (p = 0.046). In conclusion, our study showed that the rs198198 of the *PRKCB1* gene may be associated with ASD in men and with some features characteristic for the disorder.

Autism spectrum disorder (ASD) is a neurodevelopmental condition with heritability of 64%–91% (Tick et al., 2016), suggesting the presence of a strong genetic background. However, a growing amount of genetic studies show that this is a complex and genetically heterogeneous disorder and can present different patterns of inheritance and underlying genetic variants (Griesi-Oliveira & Sertié, 2017). The cases may be caused by well-known genetic factors, such as chromosomal aberrations, responsible for 5% of ASD (i.e. Prader-Willi or Angelman syndrome), copy number variants—10%–20% (i.e. submicroscopic deletions and duplications), or single- gene disorders—5% (i.e. Rett syndrome or fragile X syndrome). Such problems are typically associated with malformations and/or dysmorphic features (Ivanov et al., 2015). However, most ASD causes are idiopathic, with no clear genetic background. In such cases, the interplay between common and rare variants, both acquired de novo and parentally inherited, may explain the underlying genetic architecture of the disorder (Griesi-Oliveira & Sertié, 2017). Gene-environment interactions can also lead to epigenetic abnormalities and cause alterations in the brain anatomy and connectivity characteristic for ASD (Wiśniowiecka-Kowalnik & Nowakowska, 2019). Thus, the complex interactions between genetic, epigenetic, and environmental factors may increase the risk for disorder development (Ivanov et al., 2015).

Several polymorphisms have so far been associated with ASD, and the heritability of ASD explained by common single nucleotide polymorphisms (SNPs) was estimated from 17% to 52% (Iakoucheva et al., 2019). The data obtained by genome-wide association study (GWAS) using 2,462,046 SNPs, undertaken in 965 individuals with ASD, added support to two candidate genes previously implicated in ASD etiology, namely *PRKCB1*, and *CBLN1*. The *PRKCB1* (protein kinase C, beta 1) gene is located on chromosome 16p11 and encodes an enzyme playing an important role in signal transduction, regulation of gene expression, and control of cell division and differentiation (Philippi et al., 2005). A previous study showed that the expression of this gene was significantly down-regulated in ASD cases compared with controls (Lintas et al., 2009). The rs198198 SNP is located within an intron of the *PRKCB1* gene in a region subject to histone H3K9 modification (Jones et al., 2013).

The second SNP-rs16946931 is located in a region flanking the *CBLN1* (cerebellin 1) gene on chromosome 16q12.1. The *CBLN1* is involved in synaptogenesis and it was previously implicated in ASD (Iakoucheva et al., 2019). Based on all of these findings, we choose these two polymorphisms for the analysis of possible association with ASD in Polish population. The third analyzed polymorphism was the intron variant rs968122 located in the *KCNMB4* gene (potassium calcium-activated channel, subfamily M, regulatory beta subunit 4). The product of this gene is fundamental to the control of smooth muscle tone and neuronal excitability. Skafidas et al. (2014) found that this polymorphism highly contributed to a clinical diagnoses of ASD.

Our study aimed to verify the hypothesis that the alleles of the analyzed polymorphisms are transmitted with a different frequency between parents and ASD children and have an association with the clinical phenotype of autism spectrum disorder.

## Methods

This cohort study was conducted in accordance with STROBE guidelines. The cohort consisted of children with autism spectrum disorders and their biological parents. Three single nucleotide polymorphisms were genotyped and a transmission disequilibrium test was performed to seeking a possible relationship between the chosen polymorphisms and ASD.

### Clinical Material

The study group comprised 206 Caucasian children with autistic spectrum disorder without known coupling and their biological parents. Among the recruited patients were 162 males (78,64%) and 44 (21,36%) females. The mean age of patients at the time of diagnosis was 7,26 ± 2,72. Children with ASD and their biological parents were inhabitants of Upper Silesia (southwestern region of Poland) and they were recruited in Department of Pediatric Neurology (John Paul II Upper Silesian Child Health Centre, Katowice) and Child Development Support Center and Psychiatric Daily Ward for Children and Adolescents in Gliwice, between 2016 and 2019. The diagnosis of autism was established by a psychiatrist using ADOS-2 protocol (Autism Diagnosis Observation Schedule) as the gold standard observational instrument (Kanne et al., 2008). The inclusion criteria were: 3–12 years of age and meeting the criteria for ASD. To get a homogeneous group of patients (which could be defined as the „non-syndromic autism” or “pure autism group”) strict exclusion criteria have been applied like the occurrence of related problems such as epilepsy, intellectual disability, and other genetic and neurological diseases.

The possible associations of SNPs (genotypes and alleles) with clinical data were studied. Associations were sought between polymorphic variants and the occurrence of perinatal trauma, infant behavior in terms of self-regulation including observation of whether the infant is calm or restless, motor development/acquisition of developmental milestones such as initiation of quadruped, and independent gait; assessment of muscle tension. In the case of communication development, questions were asked about eye contact, when the first words and complete sentences appeared, whether there was developmental regression, the occurrence of compulsive, ritualistic, or self-aggressive behavior. In addition, sensory impairments including hearing and vision and the presence of self-regulatory skills including sleep, falling asleep, and the need for specialist care were considered.

The study protocol was approved by the Ethics Committee of the Medical University of Silesia in Katowice no. of application KNW/022/KB1/27/1/15. Written consents were submitted by the parents of the patients. The methods used in the study were following the Helsinki Declaration of 1975 and its further revisions.

### Genetic Analysis

Genomic DNA was extracted from peripheral leukocytes using the MasterPure™ genomic DNA purification kit (Epicentre Technologies). Polymorphisms of *KCNB4* (rs968122), *PRKCB* (rs198198), and *CBLN1* (rs16946931) genes were genotyped using specific TaqMan® Pre-designed SNP Genotyping Assay Kit (Applied Biosystems, Foster City, CA, USA). The 20 μL reaction mix consisted of the following: 1 μL template DNA (15 ng/μL), 10 μL TaqMan Genotyping Master Mix (cat. number 4371355), 1 μL probe (TaqMan Pre-designed SNP Genotyping Assay), and 8 μL deionized water. The probe was diluted in TE buffer (10 mM Tris–HCl (pH 8.0), 0.1 mM EDTA) (1:1) before the reaction. PCR was performed according to the manufacturer’s specifications. Genotyping was performed using a Roche LightCycler®96. Genotyping accuracy was checked by regenotyping 15% of the samples, and the reproducibility of the results was 100%.

### Statistical Analysis

Allele frequencies were estimated based on the genotype distribution. Hardy–Weinberg equilibrium in all groups was tested by the χ^2^ test. Normality of distribution of quantitative data was assessed by the Shapiro–Wilk test and then a comparison was performed by the Mann–Whitney U test (for variables with non-normal distribution) or the student’s t- test (for variables with normal distribution). The transmission/disequilibrium test (TDT) was used for the analysis of a possible relationship between the chosen polymorphism and ASD. TDT is based on the analysis of the transmission of specific alleles from heterozygous parents to their affected children. Only informative trios were used for calculating transmitted or non-transmitted alleles. The informative trio was defined as a family with at least one heterozygous parent. Only in such informative families, it was possible to deduce which alleles had been transmitted from parent to child. Transmission of a particular allele to a child is expected to be 50% if there is no association between the allele and the disorder. An excess transmission to the offspring is expected if the allele is associated with an increased risk of disorder. The frequencies of the transmitted alleles observed in the study were compared with the expected frequencies using the χ^2^ test. Association analysis between genetic factors and clinical features of autistic children and their parents was also performed using the *χ*^2^ test and Yates exact test when the number of subjects was lower than 10 in the 2 × 2 contingency table. The Bonferroni correction was used to eliminate the increased risk of a type I error when making statistical tests for multiple comparisons.

## Results

Alleles and genotypes distribution of all analyzed polymorphisms was consistent with the Hardy–Weinberg equilibrium. There were 206 full families (both parents and a child) analyzed in the transmission/disequilibrium test. Clinical characteristics of the study group consisted of motor development of observation, communication, and compulsive, ritualistic, or self-aggressive behavior (Table 1).

**Table 1:** Clinical characteristics of the study group

	n (%)
Perinatal trauma	8 (3.88)
Infant was excessively calm	34 (16.50)
Infant was restless	75 (36.41)
Abnormal motor development	78 (37.86)
Hypertonia	36 (17.48)
Hypotonia	66 (32.04)
Regression in communication	77 (37.38)
Impairment in eye contact	154 (74.76)
Occurrence of compulsive, ritualistic behaviour	140 (67.96)
Occurrence of self-aggressive behaviour	77 (37.38)
Hearing impairments	31 (15.05)
Vision impairments	53 (25.73)
Sleep impairments	95 (46.12)
Falling asleep impairments	58 (28.16)
Mobility/vitality	143 (68.75)

	Mean ± SD (months)
Initiation of quadruped	7.93 ± 3.70
Initiation of independent gait	13.55 ± 3.31
Time of appearance of first words	17.02 ± 12.07
Time of appearance of first complete sentences	39.73 ± 23.57

We observed that the T-allele of the rs198198 polymorphism of the *PRKCB1* gene was more often transmitted to affected children (54.9% vs. 45.1%), however, the difference was not statistically significant (χ^2^ = 3.56; p = 0.060; Table 2).

**Table 2:** Transmission/disequilibrium test for the entire group, n = 206

Allele	Transmitted n (%)	Not transmitted n (%)	χ^2^; *p*
rs198198	n informative trios = 141		
A	82 (45.1)	100 (54.9)	3.56; 0.060
T	100 (54.9)	82 (45.1)	3.56; 0.060
rs968122	n informative trios = 127		
C	79 (50.6)	77 (49.4)	0.05; 0.821
T	77 (49.4)	79 (50.6)	0.05; 0.821
rs16946931	n informative trios = 85		
T	48 (49.5)	49 (50.5)	0.02; 0.886
C	49 (50.5)	48 (49.5)	0.02; 0.886

After dividing the group of patients into subgroups according to sex, a significant difference was observed in the transmission of rs198198 alleles in the male subgroup (57.4% vs 42.6%, χ^2^ = 6.54, p = 0.010, Table 3). This difference remained statistically significant after taking into account the Bonferroni correction for multiple comparisons (p < 0.025). There was no such dependency in the female subgroup or in another polymorphism in both subgroups.

**Table 3:** Transmission/disequilibrium test for rs198198 polymorphism for male (n = 162) and female (n = 44) patients

Allele	Transmitted n (%)	Not transmitted n (%)	χ^2^; *p*
	Boys n informative trios = 112		
A	63 (42.6)	85 (57.4)	6.54; 0.010*
T	85 (57.4)	63 (42.6)	6.54; 0.010*
	Girls n informative trios = 29		
A	19 (55.9)	15 (44.1)	0.94; 0.332
T	15 (44.1)	19 (55.9)	0.94; 0.332

*Statistically significant difference after Bonferroni correction

In the entire study group, there were also 44 patients who had affected siblings, what could suggest a stronger genetic background of the disorder in such families. However, subgroup analysis did not show any statistically significant differences in allele transmission neither in patients who had nor who had no affected siblings.

Genotype–phenotype correlation analysis showed that the T allele carrier state was statistically associated with hypotonia in contrast to AA homozygosity (p = 0.048). On the other hand, in the female subgroup, the T-allele carriers more often showed more mobile/ vital behavior (p = 0.046; Table 4). Both these results showed statistical significance only in the univariate model and were not significant after correction for multiple comparisons (p > 0.025).

**Table 4:** Association between rs198198 and hypotonia or mobility/ vitality

Genotype frequency of rs198198 polymorphism and hypotonia in the male subgroup				
rs198198 variant	Hypotonia			χ^2^; *p*
	+	–		
AT + TT	39	63	vs. AA	3.90; 0.048*
AA	10	36		

Genotype frequency of rs198198 polymorphism and mobility/ vitality in the female subgroup				
rs198198 variant	Mobility/ vitality			χ^2^ (Yates exact; *p*)
	+	–		
AT + TT	19	5	vs. AA	5.27; 0.046*
AA	8	10		

*Statistically significant difference

## Discussion

In the present study, we showed the association between the rs198198 polymorphism of the *PRKCB1* gene and ASD in the male subgroup of patients, which constituted the majority of the analyzed group. The preferential transmission of T-allele from parents to affected children was observed. The difference in allele transmission was not significant in the entire group and in the subgroup of female patients. There were no associations between the next two analyzed polymorphisms and the disorder.

The rs198198 polymorphism was chosen for our analysis based on GWA study (Jones et al., 2013). Although in that study, no SNP association reached genome-wide significance, it added support to two positional candidate genes, *PRKCB1* (rs198198) and *CBLN1* (rs16946931). The *PRKCB1* gene encodes PRKCB enzyme, a member of the protein kinase C family. In some studies, the *PRKCB1* gene haplotypes were significantly associated with autism (Lintas et al., 2009; Philippi et al., 2005) but none of these analyzed haplotypes contained the rs198198 polymorphism. Additionally, there was shown the decreased *PRKCB1* gene expression in ASD patients compared to controls (Jones et al., 2013). The interesting finding of our work was that a strong association between the rs198198 polymorphism and ASD was observed only in the male subgroup. There is the common knowledge that the ratio of male to female in patients with ASD is about four to one or three to one in higher- functioning patients, which was confirmed also in our group. Some studies suggest that the prenatal and perinatal environment may play a role in the etiology of ASD. Particularly, testosterone exposure seems to influence cognitive and psychological brain development, and high levels of this hormone during early development might be a risk factor for ASD (Gamez-Del-Estal et al., 2014). Testosterone acts by binding to the androgen receptor (AR), a nuclear receptor, which regulates the expression of many genes. During androgen-dependent gene activation, histone demethylases are involved in the control of gene expression. It has been reported, that phosphorylation of histone H3 by PRKCB1 prevents from demethylation of histone H3, influencing in such a way androgen receptor-dependent gene activation (Metzger et al., 2010).

There is no experimental evidence that the rs198198 SNP is a functional variant but on the basis of bioinformatic evaluation (ENCODE data through Regulome DB and the UCSC Genome browser) Jones et al., (2013) showed that the rs198198 SNP may have possible functional potential. The T-allele was embedded within a near-consensus CCAAT/enhancer- binding protein (C/EBP) gamma binding site whereas the minor allele (A) was predicted to ablate C/EBP gamma binding. The evidence from ENCODE ChIP-seq data indicated that the rs198198 is located in a region subject to histone H3K9 modification. Thus, this potential functional significance of the rs198198 polymorphism could explain the sex-dependent association of this polymorphism with ASD. Additionally, the rs198198 SNP may be in linkage disequilibrium with other functional polymorphisms, what seems to be confirmed by Ma et al. (2010).

In the current study, we also showed an association between the T-allele carrier state of the rs198198 polymorphism of the *PRKCB1* gene and hypotonia in male patients with ASD. However, this result should be taken with caution as the difference was not significant after the Bonferroni correction. Motor delays, low muscle tone in early development are commonly reported in children with ASD (Serdarevic et al., 2017). According to many authors, neuromotor function during infancy is an important early indicator of central nervous system development. Recent and previously published data suggest that structural brain abnormalities in autism occur within regions of the brain involved in the movement, including the frontal lobe and the cerebellum. Some studies also suggest that genes expressed in brain regions that control autistic behavior can also affect motor behaviors (Hashem et al., 2020; Mostofsky et al., 2009)*.* Thus, we can assume that the *PRKCB1* gene, being particularly expressed in the hippocampus, striatum, suprachiasmatic nucleus, and cerebellar granule cells, can affect both motor skills, as well as impaired communicative and social development in children with autism (Lintas et al., 2009). The role of the *PRKCB1* gene and its polymorphism in conditioning certain motor features seem to be confirmed by the observation that the T allele carrier state was associated with higher mobility and vitality of our female patients in univariate model.

The limitation of our study is a small subgroup of female patients with ASD, which may result from the ratio of ASD patients in the population. Despite the strong association, in the GWAS research, between the chosen polymorphism and ASD, there is a lack of detailed information about its clinical or functional impact. The small size of the group could also have an impact on the results of multiple comparisons, however the observed trends seem to be explainable in the light of the subject literature. Further studies should be performed on this aspect.

In conclusion, our study seems to give support to the hypothesis that the rs198198 polymorphism of the *PRKCB1* gene may affect the development of children’s brains via androgen receptor-dependent regulation of some genes, what can influence the appearance of some features characteristic of ASD.
